# Cephalic Version

**Published:** 1859-01

**Authors:** J. H. Brower

**Affiliations:** Lawrenceburgh, Ind.


ARTICLE II.

### CEPHALIC VERSION.

BY J. H. BROWER, M.D., OF LAWRENCEBURGH, IND.

At one of the annual meetings of the Ohio State Medical Society, I had the pleasure to listen to the reading of a prize essay upon "Difficult Labors, and their mode of treatment," by Prof. M. B. Wright, of Cincinnati, which, for justness and originality of views, and correct modes of treatment, is, in my opinion, entitled to the favorable notice of the profession, and places its author in an exalted position, as a close and accurate thinker and distinguished obstetrician. The subject of shoulder presentation formed the theme of the essay, and I propose to give a condensed *resume* of the most prominent and interesting points discussed in it.

Although presentation of the right or left shoulder of the fœtus at the brim of the pelvis is of comparatively rare occurrence, yet most obstetricians of extended practice have occasionally met with instances of it; and as the motto of every accomplished accoucheur should be "semper paratus," their comparative infrequency affords no justification for the plea of ignorance, or want of tact and adroitness in the necessary manipulations. Distinguished obstetricians, of high authority with the profession, have differed widely in their views and advice, as to the best mode of treatment of such presentations. Denman, among the most reliable, strongly avers, that as nature is adequate to the accomplishment of her own wise designs, they may be left with safety to the spontaneous action of the uterus, which, by its alternate contractions, causes the shoulder presenting to be moved from its position until the head or breech occupied its place, and become the fixed presentation. That this "spontaneous evolution of the fœtus," so called by Denman, may and has occurred, is not doubted; but we opine, that in the present state of our knowledge, no conscientious or competent physician will calmly stand idle and witness the prolonged agonies and protracted sufferings of a patient, during spontaneous expulsion at the full term of gestation.

Dr. Douglas, of Dublin, the accuracy of whose views has been acknowledged by more recent writers, gives a different explanation of the manner in which spontaneous delivery is accomplished in shoulder presentations — which is beyond doubt more correct, to which the expression, "*spontaneous expulsion*," may be more correctly applied.

Dr. Churchill says, "the head and the shoulder depressed in the pelvis are fixed, and the remainder of the body, doubled up, is inch by inch forced into the pelvis and through the external parts, until all below the arm is expelled, leaving the case to terminate as a breach or foot presentation." At no part of the process is the arm at all retracted, but if moved at all, it is still farther protruded. A labor of this description requires unparalleled voluntary efforts, and necessarily involves extreme suffering of body and mind, and extreme danger during or soon after delivery, or incurable injury and ruined general health. The practical question is, shall the physician stand idle and hope for delivery by the intense and protracted suffering of his patient by either of these modes of *spontaneous evolution* or *spontaneous expulsion*, or by a recourse to a more humane and scientific procedure, accomplish a delivery, "cito, tute, et jacunde?"

Among American authorities, Dr. Dewees, after admitting that spontaneous evolution has never occurred in his practice, says, "I should therefore recommend waiting for this spontaneous evolution, whenever turning (podalic version) forbade the hope of saving the child, provided the labor be not complicated by some accidental circumstances, such as flooding, convulsions, syncope," etc. He quotes Dr. Merriman as saying, "the occurrence of the spontaneous evolution has been comparatively so rare that no man would be justifiable in simply relying upon it. The knowledge that it has sometimes happened may, indeed, under some circumstances of extreme resistance to the passage of the hand into the uterus, reconcile us to the delay which I have recommended, but we should never allow it to operate upon our minds so as to induce us to neglect the proper means and proper time of turning, when we have it in our power. It is the duty of the accoucheur on all occasions to give nature every possible opportunity of exerting herself for the relief of the patient; but it is equally his duty, when nature becomes embarrassed and oppressed, to interpose the timely assistance of art, lest nature being compelled to relinquish the task, the patient shall fall a sacrifice to the delay."

Dr. Davis, in his Elements of Operative Midwifery, concludes some observations on this subject with the following words: "If, therefore, we suppose the child to be already dead, or the circumstances of the labor to be such as to make it impracticable to bring it into the world alive by means of turning (podalic version), or even to perform that operation at all without exposing the mother to extreme danger, it would then, in my opinion, be the unquestionable duty of the practitioner to effect the delivery by embryotomy." According to Velpeau, only twelve children were alive after spontaneous expulsion out of 137 labors.

From these extremely vague and even contradictory views of the proper management of shoulder presentations, it will be readily perceived how unsatisfactory are the authorities cited in deciding upon their relative merits. The questions now arise,—

1st. In what manner can a change of presentation be accomplished most easily and successfully?

2d. What mode of proceeding will prove most favorable for the mother?

3d. How may the life of the child be best preserved?

4th. Can any mode of delivery be relied on exclusively?

Two modes of delivery, besides those already alluded to, have been described by obstetrical writers, viz: *podalic version*, or turning by the feet, and *cephalic version*, or turning by the head. Strictly speaking, however, cephalic *version* is not performed, if version and turning are used as synonymous terms.

When to expedite labor we substitute the head for the shoulder, we simply remove one part that the other may occupy its place. Dr. Dewees, and Prof. Bedford in his translation of Dr. Chailly's Treatise on Midwifery, are silent upon the subject of cephalic version in shoulder presentations. Prof. Meigs remarks, "It may be that those old practitioners of the days of Queen Elizabeth may have sometimes succeeded by pushing up the presenting shoulder in getting the head at last to come to the strait again, but such an event in any case appears to me most improbable." Prof. Heuston says, "The practitioner will experience great difficulty, and most likely fail, in attempting to bring down the head in a favorable position when the shoulder presents. I am satisfied, from considerable experience, that when the arm, shoulder, breast, back, or side is the presenting part, it is better to bring down the feet at once while the condition of the uterus is favorable for turning, than to waste time in attempting to restore the head." Prof. Miller uses the following language: "Cephalic version has but few advocates at the present day, and is confessedly applicable to such a limited number of cases that it is scarcely worthy of our formal consideration. For this reason, and because I have no experience of it, I shall confine my observations to podalic version. Again, it is manifest that all attempts forcibly to pass the hand between a powerfully contracted uterus and the fœtus must be extremely painful, and may cause fatal rupture of the organ. The only resort is mutilation of the child, either by eviscerating its trunk, to enable the operator to extract it doubled upon itself, in imitation of the natural process of duplication, or by duplicating it in order that the body and head may be separately extracted." Dr. Churchill says, "Should version by the feet be impracticable, we must open the chest of the child and eviscerate, after which it may be extracted by the crotchet." Dr. Ramsbotham claims that of the three modes of treatment, that of raising the shoulder and bringing down the head would be the safest for the child, because there would then be little chance of pressure on the funis." The opinions of Velpeau are thus expressed: "Cephalic version may be attempted,—1st, in a well-formed pelvis, where no other accident has happened except a vicious position of the fœtus, and the head is found in an inclined position in the vicinity of the strait; 2d, in presentations of the shoulder, back, or anterior part of the thorax, *provided the arm is not prolapsed*, and the uterus not too much contracted; lastly, it seems proper to try it whenever the feet are further removed from the strait than the head is, and where it is probable the labor would terminate spontaneously if the head were at the strait." Chailly admits that delivery by cephalic version "will be as happy for the infant and mother as if the vertex had originally presented," but advises it only before the rupture of the membranes.

After this hasty outline of the views entertained by several reliable authorities, Dr. Wright proceeds to detail his own treatment of several cases of shoulder presentation, five in number, in all of which the membranes had been ruptured, and in which delivery was speedily effected by cephalic version, with entire safety to the mothers and life to two of the five children. Dr. W.'s mode of manipulation was as follows: the patient being on her back across the bed in the usual position for turning, the prolapsed arm when descended having been returned by introducing the right hand, the shoulder and thorax were grasped and the body thus pushed upwards, and laterally, by which manœuvre, the head was brought near the axis of the pelvis; he then relinquished his hold of the body, and grasping the occiput, brought it down so as enable the head to engage.

The result of five cases of shoulder presentations which have come under my own notice in an extended obstetric practice of thirty-five years, fully confirms the views of Prof. Wright. In one which occurred at an early period of my practice, instructed by the precepts of Denman and Francis, the case was left to the hazards of spontaneous evolution; the fœtus was, indeed, at length involved, but at the expense of such prolonged and agonizing torture, and such utter ruin of health of the mother, as to impress me most strongly with the truth that although *a meddlesome midwifery is bad*, yet that tardy and irresolute action is often followed by a greater amount of evil. In the second case, the presentation was distinctly made out immediately upon the rupture of the membranes, and I determined at once to attempt to bring down the feet and thus effect the delivery. By the aid of bleeding, opium and tart. antim., this was effected, though with great difficulty and at the expense of the life of the child, which was destroyed by pressure upon the funis during the descent of the head. Two years subsequent to this, the same lady demanded my services, having had strong pains for two or three hours prior to my arrival. I found the left arm projecting from the vagina, the water escaped, and the os uteri fully dilated. Having in the interim become acquainted with Velpeau's opinions, I determined to attempt cephalic version, and after returning the prolapsed arm by bending the forearm until the hand was directed to the upper part of the vagina, and then pushed up until it ascended above the brim of the pelvis, the head was found resting on the right side of the pelvis, with the occiput in front and the face toward the promonotory of the sacrum; by steadily applying gentle force to the shoulder, laterally and upwards, during the intervals of expulsive effort, the body was moved until the breech had ascended to the fundus of the uterus, the face and vertex gradually descending until it became the presenting part. As, during the absence of uterine contraction, the vertex seemed disposed to return to its original malposition. I gave infus. ergot so as to induce its peculiar action in keeping up continuous contractile effort, by which means the labor soon completed with safety to both mother and child. In the third and fifth cases, in which I was concerned in consultation, laceration and rupture of the uterns resulted from imprudent attempts to bring down the feet, and the force employed in a fruitless effort to change the presentation. In the fourth case, under my own care, cephalic version was readily accomplished with the aid of the forceps, with no other accident save a slight laceration of the scalp, from peculiar difficulty in adjusting the blades of the instrument.

The results of these cases fully justifies us in declaring—

1st. That at an early period in labor, and especially if called before a rupture of the membranes, a shoulder *may* be converted into a vertex presentation more easily than turning by the feet is ordinarily performed.

2d. That although the membranes may have been long ruptured, turning by the hand can be accomplished with great facility.

3d. That delivery by cephalic version may be speedily effected after repeated and ineffectual efforts have been made to turn by the feet.

4th. That cephalic version should receive a prominent, nay, leading place as a means of expediting delivery in shoulder presentations.

The second of the questions already proposed is, What mode of proceeding will prove most favorable to the mother?

Dr. Churchill observes: "From the distance the head has to traverse in podalic version, and the difficulty of seizing the feet and of turning the child in utero, there must ever be a fearful risk of injury to the mother." From Dr. Lee's tabular views, we find that out of seventy-one cases of shoulder presentations, in which turning of the feet was resorted to, seven died from rupture and three from inflammation of the uterus. In cephalic version, the hand does not enter the cavity of the uterus, and, consequently, neither its walls nor any portion of them are forcibly pushed out. The fœtus is moved comparatively little within the uterus, the head being already near the superior strait; while in podalic version the part to be first delivered is most remote from the canal through which it must pass. In the former, the injury to the mother cannot result without great awkwardness on the part of the obstetrician, while in the other we have reason to feel surprised at the escape of injury. In turning by the feet, the hand must necessarily be moved considerably within the uterus, and often while it is contracting violently. In turning by the head there is but little, if any, direct contact of the hand within the uterus. In the one case, contusion of the uterus by the hand is to be expected; in the other there is no injury because there is no contact. Turning by the feet may occasion a severe nervous shock; not so in changing the shoulder for the head.

How may the life of the child be best preserved? is the third question to be answered.

The mortality in shoulder presentations is, doubtless, greater than in turning by the feet in all cases, which Churchill states as one in three. A timely resort to cephalic version gives to the fœtus almost as much certainty of life as if the presentation had been originally in the head, for the manœuvre amounts to but little more than in rectifying deviated head positions. In 1826, Bush gave an account of fifteen cases, in which fourteen were born living. In 1827, Ritgen collected forty-five successful cases. Rucke has had sixteen cases. Prof. Wright says, "In all the cases treated by myself, from the beginning, the children were born alive. The liability to compression of the chord and consequent death of the fœtus is in proportion to the length of the labor, or rather to the descent of the fœtus in the cavity of the pelvis. Hence, to be wholly successful, cephalic version should be performed a short time before or soon after the commencement of the second stage of labor."

Can any one mode of treating shoulder presentations be relied on exclusively?

The answer must be in the negative. Cazeaux says, "At the present day it would be improper to embrace either opinion exclusively, for some cases are better suited to the cephalic version, while there are others, on the contrary, where the pelvic one is alone practicable; consequently, both operations should be retained in practice, leaving the judgment of the accoucheur to determine the cases where the one or the other ought to be preferred." In cases of inefficient uterine action, or in exhaustion from long continued labor, in hemorrhage, convulsions, or in any case in which there may be a demand for speedy delivery, turning by the feet is to be preferred. In all cases where difficulty arises from malposition alone, or in convulsions, hemorrhage, or in prolapsus of the funis, if the uterus should be engaged in vigorous expulsive efforts, turning by the head should be selected.

Prof. Wright's manner of performing cephalic version is as follows: The patient having been placed upon her back across the bed, and with her hips near its edge—supposing the presentation to be the right shoulder, with the head in the left iliac fossa, the right hand to have been introduced into the vagina, and the arm, if prolapsed, having been placed as near as may be in its original position across the breast—the fingers are applied upon the top of a shoulder and the thumb in the opposite axilla, or on such part as will give us command of the chest, and enable us to apply a degree of lateral force. The left hand is also applied to the abdomen of the patient, over the breech of the fœtus. Lateral pressure is made upon the shoulders in such a way as to give to the body of the fœtus a curvilinear movement. At the same time, the left hand, applied as above, makes pressure so as to dislodge the breech, as it were, and move it towards the centre of the uterine cavity. The body is thus made to assume its original bent position, the points of contact with the uterus are loosened, and perhaps diminished, and the force of adhesion is in a good degree overcome. Without any direct action upon the head, it gradually approaches the superior strait, falls into the opening, and will, in all probability, adjust itself as a favorable vertex presentation. If not, the head may be acted upon as in deviated positions of the vertex, or it may be grasped, brought into the strait, and placed in correspondence with one of the oblique diameters.

It will be observed that the shoulders are not acted upon by raising them. Perhaps a slight elevation would facilitate the movement already described, or it might be better to depress them, and again, by lateral pressure, without either elevation or depression, our object might be accomplished. Pushing up the shoulders, then, does not constitute a prominent part of turning, if by pushing up is meant the mere raising the shoulders above the brim of the pelvis. The entire process of cephalic version is to be adopted in the absence of uterine contraction, or rather during the intervals of expulsive force, and as it is now a vertex presentation, we must be governed as to the manner and time of delivery by those general rules applicable to such cases.

Prof. Wright, in conclusion, remarks: "If our mode of performing cephalic version is sufficiently clear in the description already given, it will be readily distinguished from others. We claim for it great importance on the ground that it is easily executed, that the mother and fœtus receive no injury, that there is little or no danger of subsequent displacement after the vertex has been fully adjusted, that although it is most successful in recent cases, delivery may be accomplished after the membranes have been long ruptured, that it may be executed after ineffectual efforts to bring down the feet."

